# Surviving Cancer, Lacking Support: The Hidden Burden of Modern Radiation Oncology in the Treatment of Oligometastatic Disease

**DOI:** 10.3390/cancers18162584

**Published:** 2026-08-11

**Authors:** Beth Chasty, Agata Rembielak, Richard Berman, Eva Oldenburger

**Affiliations:** 1 The Christie NHS Foundation Trust, Manchester M20 4BX, UK; r.berman@nhs.net; 2 Division of Cancer Sciences, School of Medical Sciences, Faculty of Biology, Medicine and Health, The University of Manchester, Manchester M13 9PL, UK; 3 Department of Radiation Oncology, University Medical Center Leiden, 2333 ZA Leiden, The Netherlands; e.s.a.oldenburger@lumc.nl

**Keywords:** oligometastatic disease, stereotactic ablative radiotherapy (SABR), metastasis-directed therapy, supportive oncology, cancer survivorship, quality of life, precision radiation oncology

## Abstract

The management of oligometastatic disease has undergone a significant paradigm shift over the past two decades. Once considered uniformly incurable, selected patients with metastatic disease can now achieve prolonged progression-free survival, durable disease control, and, in carefully selected cases, long-term remission or cure through metastasis-directed therapies. Advances in stereotactic ablative radiotherapy (SABR), surgery, systemic therapies, and the emerging concept of Curative Oligometastatic Radiotherapy (CORT) have challenged the traditional distinction between curative and palliative treatment. Concurrent developments in imaging, including PET/CT, prostate-specific membrane antigen (PSMA) PET, whole-body MRI, and MR-guided adaptive radiotherapy (MR-linac), together with evolving biomarker research, are improving disease characterisation, refining patient selection, and treatment personalisation. As survival improves, an increasing number of patients are living with durably controlled metastatic cancer and experience long-term physical, psychological, cognitive, functional, and financial consequences of treatment. Despite these challenges, evidence-based survivorship pathways for patients with oligometastatic disease remain poorly defined. Supportive oncology is becoming an essential component of modern radiation oncology rather than an adjunct to cancer treatment. This emerging discipline focuses on optimising symptom control, minimising toxicity, and delivering structured survivorship care. Rather than being limited to end-of-life care, supportive oncology is embedded throughout the patient journey; from diagnosis and treatment selection to prehabilitation, rehabilitation, patient-reported outcome (PRO) monitoring, surveillance for late effects, multidisciplinary follow-up, and long-term survivorship. This review discusses how advances in precision radiotherapy, molecular imaging, biomarkers, and emerging treatment technologies are reshaping the management of oligometastatic disease while simultaneously creating a growing population of long-term survivors with increasingly complex supportive care needs. It highlights the expanding role of supportive oncology in the care of patients with oligometastatic disease, encompassing multidisciplinary symptom management and argues that improvements in disease control must now be matched by the development of evidence-based multidisciplinary survivorship pathways that integrate supportive oncology to optimise quality of life (QoL), functional independence, and patient-centred outcomes. Finally, this review highlights current evidence gaps and proposes future research priorities for developing evidence-based survivorship models for this rapidly expanding patient population.

## Simple Summary

The concept that metastatic disease is incurable has been challenged by advances in imaging and treatment. In the oligometastatic setting (limited sites of metastatic disease), developments in both surgery and radiotherapy have enabled long-term disease control and even cure in a growing number of cases. As a result, there is a growing population of patients living with and beyond cancer. There is a mounting need to address the services available to these patients who, despite improved survival, are often living with the long-term effects of both cancer and previous treatments. Supportive oncology, a growing specialty focused on managing the side effects of cancer and its treatment at all stages of the cancer pathway, has recognised this gap in care and aims to support patients to improve quality of life, promote survivorship care and minimise late effects.

## 1. Introduction

Historically, treatment of metastatic cancer has largely been delivered with palliative intent. In radiotherapy, this meant using short treatment courses with a relatively low dose, focusing on symptom control, maintenance of quality of life (QoL), and prevention of cancer-related complications. In contrast, high-dose curative radiotherapy has traditionally been reserved for localised disease, where treatment is directed toward eradication of all known tumour sites, aiming for cure.

However, an increasing number of patients with advanced cancer are now achieving prolonged disease control and extended survival, even in the metastatic setting. Improvements in systemic therapies, particularly targeted therapies and immunotherapy, together with advances in molecular imaging, surgical techniques, and radiotherapy, have increasingly challenged the binary distinction between curative and palliative cancer treatment across multiple tumour types, including melanoma, non-small cell lung cancer, renal cell carcinoma, prostate cancer, colorectal cancer, and breast cancer [1,2,3,4].

For some patients, metastatic disease behaves in a more indolent manner, allowing aggressive (local) treatment approaches to achieve durable remission and, in selected cases, long-term survival or even cure. Consequently, the management of metastatic cancer has undergone a major paradigm shift, particularly with the emergence of the oligometastatic disease concept. These developments have blurred the traditional boundaries between palliative and curative oncology and created a growing cohort of patients who do not fit neatly into either category.

This evolving new patient population occupies a poorly defined space within contemporary cancer care. Although their disease often remains biologically incurable, many experience prolonged periods of disease control, measured in years rather than months.

Traditional survivorship services have largely been designed for patients treated with curative intent, whereas specialist palliative care services have historically focused on patients with progressive disease and end-of-life needs. Consequently, patients living with durable controlled advanced cancer frequently exist between these two established models of care, creating a substantial gap in service provision, where neither model adequately addresses their long-term needs [5]. Follow-up for these patients traditionally centred on surveillance imaging and the detection of disease progression or recurrence, with relatively little emphasis on the systematic assessment and management of treatment-related toxicity, symptom burden, functional recovery, psychosocial well-being, or health-related quality of life.

As survival improves, however, this predominantly disease-focused approach is increasingly insufficient. Many patients undergo repeated courses of local and systemic treatment over several years, resulting in cumulative acute and late toxicities, persistent fatigue, pain, cognitive impairment, psychological distress, and reduced participation in work and daily activities. These challenges often fluctuate throughout the disease trajectory and may persist even during prolonged periods of radiological disease control. Consequently, longitudinal follow-up should extend beyond tumour surveillance alone and incorporate routine assessment of patient-reported outcomes, supportive care needs, rehabilitation requirements, and opportunities for health promotion, including exercise, nutritional support, and psychosocial interventions.

This evolving model reflects a shift from episodic, treatment-focused care towards continuous survivorship care, delivered alongside active oncological treatment. Rather than viewing supportive oncology and palliative care as services introduced only after treatment options have been exhausted, they should be integrated throughout the disease trajectory to optimise symptom management, maintain functional independence, support shared decision-making, and preserve quality of life. Such an approach requires close collaboration between different specialists, such as radiation and medical oncologists, surgeons, specialist nurses, rehabilitation physicians, physiotherapists, psychologists, dietitians, palliative care specialists, and primary care providers.

As advances in oncology treatment continue to expand the population of long-term survivors with advanced cancer, understanding how best to support these patients has become an increasingly important challenge for modern oncology. Developing evidence-based follow-up pathways will therefore be essential to deliver truly patient-centred care for individuals living with cancer.

This review examines how advances in imaging, radiotherapy, and biomarker-driven patient selection have transformed the management of oligometastatic disease and discusses the emerging role of supportive oncology in addressing the complex survivorship needs of this rapidly growing patient population, emphasising that the evolution of oligometastatic disease into a chronic survivorship condition requires a new model of multidisciplinary care.

## 2. Oligometastatic Disease

Oligometastatic disease describes an intermediate state between localised cancer and widespread (polymetastatic) disease, first introduced in 1995 by Hellman and Weichselbaum [6]. They outlined the potential for achieving cure through local treatment of metastases, which challenged the pre-existing understanding that all metastatic disease was uniformly incurable [7,8]. Commonly, this state has been described as 1–5 metastatic sites which are that can be treated safely with ablative intent; however, the definition has continued to evolve. Further research incorporating advances in imaging and biomarker discovery has generated tighter criteria for patient selection, aiming to target those who are likely to benefit from local therapy [9].

**Figure 1:**
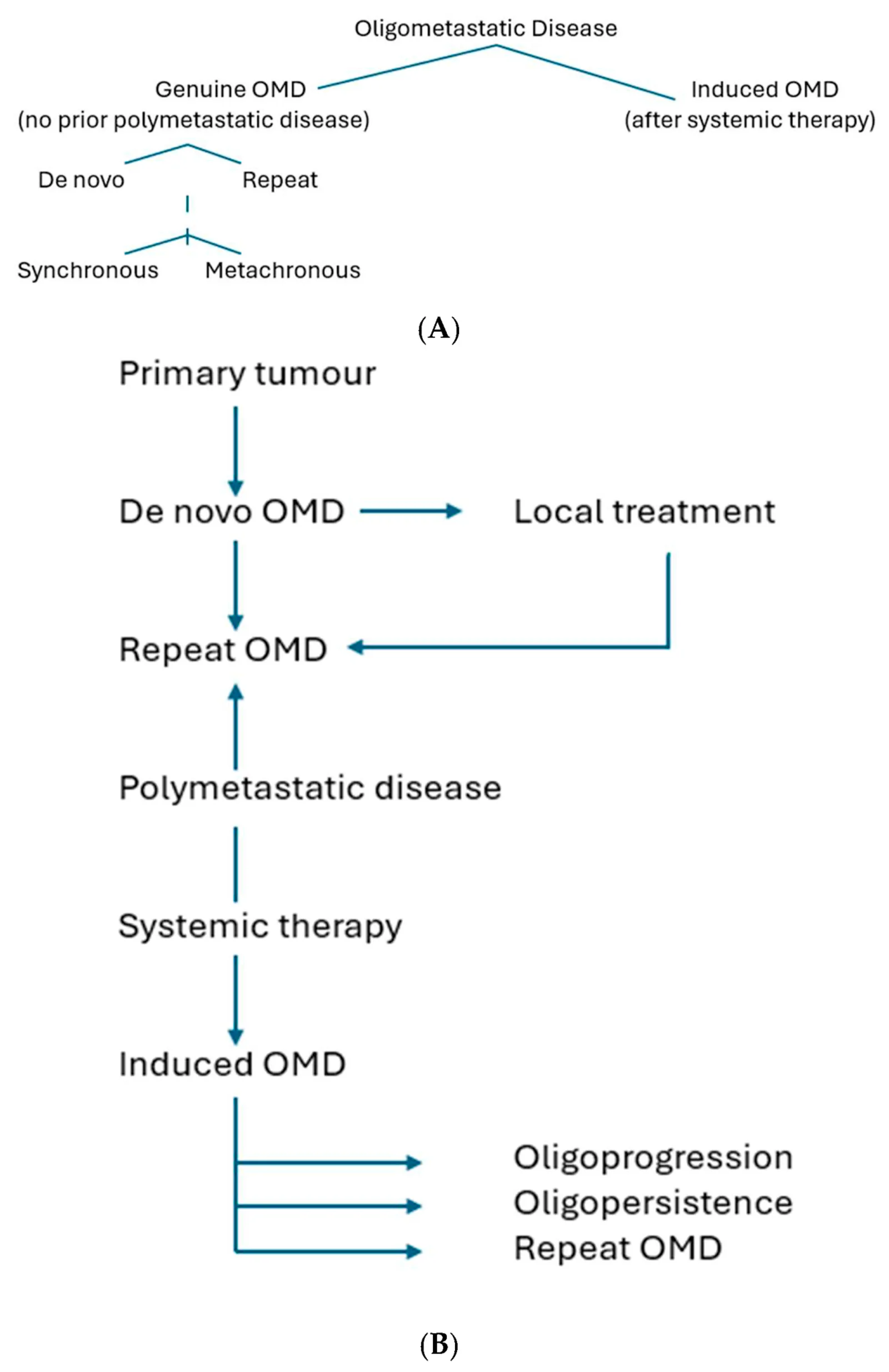
(**A**) Biological and Temporal Classification; (**B**) Dynamic Disease Evolution. OMD: oligometastatic disease. Figure based primarily on the oligometastatic the ESTRO/EORTC classification, which represents a patient’s disease state at a specific point in time. Patients may transition between categories during their disease course [10].

More recently, the European Society for Radiotherapy and Oncology (ESTRO) and the European Organisation for Research and Treatment of Cancer (EORTC) proposed a biological and temporal classification system for oligometastatic disease through an international Delphi consensus process, recognising the considerable heterogeneity within this patient population. The framework distinguishes genuine oligometastatic disease, in which patients have no prior history of polymetastatic disease, from induced oligometastatic disease, describing patients who initially presented with polymetastatic disease but, following systemic therapy, have only a limited number of residual metastatic lesions. Genuine oligometastatic disease is further subdivided into de novo oligometastatic disease, representing the first diagnosis of oligometastatic disease, and repeat oligometastatic disease, referring to patients with recurrent episodes of oligometastatic disease. De novo oligometastatic disease may be further classified as synchronous, when metastases are identified at the time of primary diagnosis, or metachronous, when they develop following an interval after initial diagnosis. The consensus also incorporates the clinical context of metastatic progression, distinguishing oligorecurrence, oligoprogression, and oligopersistence based on whether oligometastatic disease is detected during a treatment-free interval or during active systemic therapy, and whether metastatic lesions are progressing on contemporary imaging. Importantly, this classification represents a patient’s disease state at a single point in time rather than a fixed disease entity. Patients may transition between different oligometastatic states throughout the disease course, for example from de novo to repeat oligometastatic disease following local treatment, or from polymetastatic to induced oligometastatic disease after systemic therapy, highlighting the dynamic nature of metastatic evolution [10,11]. A schematic summary of this classification is provided in Figure 1.

Niibe et al. additionally described the concept of oligorecurrence, characterised by metachronous oligometastases with a controlled primary lesion, highlighting the importance of the status of the primary lesion in determining prognosis [12,13,14]. Other clinically relevant states include oligoprogression, where progression occurs in a limited number of lesions during systemic therapy, and oligopersistence, where treatment of initially polymetastatic disease results in a residual low-volume metastatic state [15,16].

These evolving classifications have important therapeutic implications, reflecting the paradigm shift from historically palliative local treatment of metastatic disease towards metastasis-directed therapy with curative or disease-modifying intent in carefully selected patients. In de novo and repeat oligometastatic disease, the primary objective is eradication of all detectable disease through comprehensive local treatment, typically combined with systemic therapy to maximise long-term disease control and, in some patients, achieve durable remission or cure. In contrast, treatment of induced oligometastatic disease complements ongoing systemic therapy, with local ablative treatment used to consolidate residual disease, restore disease control in oligoprogression, or deepen treatment response in oligopersistence. As patients may transition between different oligometastatic states over the course of their disease, treatment strategies should be adapted dynamically according to disease evolution, prior therapies, and the balance between local and systemic treatment.

Underpinning this therapeutic approach is the growing recognition that oligometastatic disease may represent a biologically distinct disease state rather than simply an intermediate stage between localised and widespread metastatic disease. Emerging translational evidence suggests that oligometastatic tumours may possess reduced metastatic potential, slower growth kinetics, and greater susceptibility to local ablative therapies than polymetastatic disease [17,18]. However, the biology of oligometastatic disease is likely heterogeneous and influenced by tumour histology, molecular subtype, and site of origin. Oligometastatic presentations are relatively common in malignancies such as prostate, colorectal cancer, and HCC but have rarely been reported in small cell lung cancer [17]. These observations suggest that the propensity to develop an oligometastatic phenotype varies across tumour types, highlighting the need for improved biological biomarkers to identify patients most likely to benefit from metastasis-directed treatment and to refine patient selection beyond anatomical disease burden alone [19].

## 3. Imaging

High-quality multimodality imaging remains the cornerstone of patient selection and the current gold standard for staging, treatment selection, and assessment of disease extent. While molecular biomarkers have considerable potential to improve risk stratification and identify patients most likely to benefit from metastasis-directed therapy, they are not yet sufficiently validated for routine clinical practice and are therefore likely to complement, rather than replace, advanced imaging in future precision oncology strategies [20].

Modern imaging techniques are essential for accurate staging, assessment of disease burden, evaluation of treatment response, and radiotherapy planning [21,22]. ^18^F-FDG-PET/CT is the preferred functional imaging modality for staging patients with non-small cell lung cancer, colorectal cancer, and breast cancer, while liver MRI provides additional sensitivity for detecting hepatic metastases, particularly in colorectal cancer. ^68^Ga-PSMA PET/CT has become the imaging standard for prostate cancer because of its superior sensitivity and specificity for detecting low-volume nodal and distant metastatic disease compared with conventional imaging [20]. Whole-body MRI offers an alternative radiation-free staging modality with excellent soft tissue contrast, which has gained increasing clinical relevance in the evaluation of several malignancies, most notably prostate cancer, lymphoma, and multiple myeloma, but also melanoma, breast, ovarian, colorectal, and lung carcinomas.

The increasing sensitivity of contemporary imaging has substantially expanded the number of patients identified with oligometastatic disease and consequently those considered eligible for metastasis-directed therapies. However, this has also introduced important challenges, including stage migration (the “Will Rogers phenomenon”), the detection of lesions with uncertain clinical significance, and potential differences in disease classification depending on the imaging modality employed. These factors emphasise that imaging findings should always be interpreted alongside tumour biology, disease kinetics, and patient-related factors when selecting candidates for local ablative treatment. Furthermore, emerging evidence suggests that traditional surveillance imaging schedules, primarily designed to detect local recurrence, may not be optimised for identifying oligometastatic relapses, which may occur several years following primary treatment [23]. Looking forward, integration of advanced imaging with molecular biomarkers, radiomics, and artificial intelligence may enable more precise prediction of disease behaviour, personalised surveillance strategies, and improved treatment selection. However, robust prospective validation and standardisation are required before these approaches can be routinely implemented in clinical practice.

Taken together, advanced imaging is becoming indispensable for identifying patients with oligometastatic disease, although its greatest value will likely emerge when integrated with molecular biomarkers rather than used in isolation.

## 4. Biomarkers

Despite advances in imaging and disease classification, selecting patients most likely to benefit from metastasis-directed therapy (MDT) remains one of the greatest challenges in the management of oligometastatic disease. Current selection relies predominantly on clinical and radiological criteria, which may fail to identify patients with occult micrometastatic disease who are unlikely to achieve durable benefit from local ablative treatment [10]. Consequently, robust biomarkers are needed to distinguish patients with a truly limited metastatic phenotype from those with biologically aggressive disease, thereby avoiding unnecessary local therapies and delays to more appropriate systemic treatment while improving patient selection for curative-intent interventions [24].

Several biomarker strategies are currently under investigation. Liquid biopsy approaches, including circulating tumour DNA (ctDNA), circulating tumour cells (CTCs), and circulating microRNAs, offer the potential to detect minimal residual disease and quantify metastatic burden through minimally invasive serial sampling. Molecular profiling, including next-generation sequencing, actionable genomic alterations, tumour mutational burden (TMB), and immune-related gene signatures, may further identify tumour subgroups with distinct metastatic behaviour and therapeutic vulnerabilities. In parallel, advances in radiomics and radiogenomics seek to extract quantitative imaging features that reflect tumour phenotype and integrate these with genomic data to improve risk stratification and treatment prediction [19,25,26].

Although these approaches have demonstrated encouraging prognostic and predictive value in retrospective analyses and early-phase studies, several important limitations remain. Most candidate biomarkers have been evaluated in small, heterogeneous cohorts, often within single tumour types, using differing analytical platforms and non-standardised thresholds. Consequently, few biomarkers have undergone prospective validation, and none are currently recommended for routine selection of patients for metastasis-directed therapy. The lack of standardisation and external validation remains a major barrier to clinical implementation.

Future progress is therefore likely to depend on integrating biological biomarkers with advanced imaging, clinical risk factors, and disease classification within prospective biomarker-driven clinical trials. Such multimodal approaches may enable a shift from the current anatomy-based definition of oligometastatic disease towards a biologically informed framework, facilitating more personalised treatment strategies and improving the balance between therapeutic benefit and treatment-related burden.

Although no biomarker is currently ready for routine clinical use, integrating biological and imaging markers represents one of the most promising strategies for refining patient selection in the coming decade.

## 5. Treatment Strategies

Advances in imaging, systemic therapy, and local ablative techniques have fundamentally changed the management of oligometastatic disease. Rather than applying a uniform treatment strategy, management is increasingly individualised according to the biological and clinical state of oligometastatic disease. Disease classification (de novo, repeat, induced, oligorecurrence, oligoprogression, or oligopersistence) has important therapeutic implications and should be considered alongside tumour biology, metastatic burden, expected survival, previous treatments, patient fitness, and patient preferences.

While patients with de novo or repeat oligometastatic disease could be considered for comprehensive metastasis-directed therapy with curative or durable disease-control intent, local ablative therapy in oligoprogression aims to eradicate resistant lesions while preserving the benefit of effective systemic therapy. Conversely, in induced oligometastatic disease, local treatment is primarily used to consolidate residual disease following systemic response rather than replace systemic therapy. These increasingly complex treatment decisions require multidisciplinary discussion to optimise oncological outcomes while minimising treatment-related toxicity and preserving QoL.

### SABR

Surgical metastasectomy has historically represented the standard local treatment approach; however, stereotactic ablative radiotherapy (SABR) has increasingly emerged as a non-invasive alternative capable of delivering highly conformal, ablative radiation doses with excellent local control and acceptable toxicity profiles [27,28]. The growing use of SABR positions radiation oncologists as key figures in this oncological area [29]. SABR offers several practical advantages, including shorter treatment duration, reduced recovery time, and applicability in patients who are medically inoperable or have anatomically challenging lesions [29].

The landmark SABR-COMET phase II randomised trial represented a major milestone in the field and demonstrated significant improvements in overall survival and progression-free survival following SABR in patients with up to five metastatic lesions across multiple tumour types [30]. Long-term follow-up demonstrated a 22-month improvement in median overall survival, with a 5-year overall survival rate of 42% in the SABR arm compared with 17% in the control arm [31]. Additional randomised studies, including ORIOLE in prostate cancer and Gomez et al. in oligometastatic non-small cell lung cancer, have further supported the role of local consolidative therapy in improving disease control and delaying progression [32,33]. Although these trials have fundamentally changed the management of oligometastatic disease, several important limitations should be acknowledged. Most studies enrolled relatively small, highly selected patient populations, included heterogeneous tumour types, and differed in definitions of oligometastatic disease, limiting the generalisability of their findings. Furthermore, evidence supporting an overall survival benefit remains strongest for selected patient groups, while the optimal timing of SABR, integration with systemic therapies, and patient selection criteria continue to evolve. An overview of these SABR trials is reported in Table 1.

Collectively, these studies established SABR as one of the principal metastasis-directed treatment modalities for patients with oligometastatic disease. However, questions remain regarding optimal patient selection, integration with modern systemic therapies, and long-term survivorship outcomes. Furthermore, SABR should not be considered a replacement for surgery in all patients. Comparative studies suggest that the relative benefits of surgery and SABR vary according to tumour histology, lesion location, patient fitness, and treatment goals. For example, Wang et al. reported superior overall survival following surgical resection compared with SABR for colorectal oligometastases, although SABR achieved comparable local control with lower early morbidity and better short-term QoL [38]. Such findings reinforce the importance of individualised treatment selection through multidisciplinary decision-making and highlight that treatment outcomes should not be evaluated solely through survival metrics but also through patient-reported outcomes, toxicity, and long-term QoL [39].

## 6. Emerging Radiotherapy and Imaging Techniques

Although SABR currently represents the best-supported local treatment modality for oligometastatic disease, several other radiotherapy technologies could potentially be applied to further improve the therapeutic ratio by increasing treatment precision, reducing toxicity, or enabling safer retreatment. However, unlike SABR, most of these technologies are supported predominantly by dosimetric studies, retrospective series, or early-phase clinical trials. Consequently, their routine adoption remains limited while prospective comparative evidence continues to evolve.

### 6.1. Proton Beam

Proton beam therapy has demonstrated promising local control across multiple oligometastatic tumour sites, with the physical properties of the Bragg peak enabling reduced dose to surrounding organs at risk compared with conventional photon radiotherapy [40,41,42]. This may be particularly advantageous in anatomically challenging sites, such as sternal or liver metastases, where reducing dose to adjacent critical structures may improve tolerability and preserve future treatment options and reduce the cumulative radiation burden in patients who may require multiple courses of local therapy during prolonged survival [42,43,44]. Such dosimetric advantages may be particularly relevant in oligometastatic disease, where long-term survivors are at greater risk of cumulative treatment-related toxicity and late effects.

Nevertheless, current evidence demonstrating clinical superiority over contemporary photon-based SABR is lacking. Most published data comprise retrospective series and dosimetric comparisons rather than randomised trials. Combined with substantial infrastructure costs and reimbursement challenges, as well as limited geographical availability, currently restrict the use of proton therapy.

### 6.2. Flash Radiotherapy

By delivering radiation at ultra-high dose rates (>40 Gy/s), FLASH radiotherapy appears to preferentially spare normal tissues while maintaining tumour control, potentially improving the therapeutic ratio of ablative radiotherapy [45,46,47]. Although frequently highlighted as a potentially transformative technology, FLASH radiotherapy remains at an early stage of clinical development. The only prospective clinical evidence (FAST-01) evaluated palliative bone metastases rather than oligometastatic disease [48].

Consequently, while FLASH has been highlighted as a technology which could provide benefit in the oligometastatic setting, its application requires prospective validation studies as clinical experience with FLASH remains extremely limited [46,49].

### 6.3. Spatially Fractionated/GRID/Lattice Radiotherapy

Similarly, spatially fractionated radiotherapy techniques, including GRID and lattice radiotherapy, use highly heterogeneous dose distributions to improve normal tissue sparing while maintaining ablative intratumoural doses [50,51]. These approaches may offer particular benefit in bulky oligometastases, re-irradiation settings, or lesions adjacent to critical organs where conventional SABR is challenging [50,52]. Their potential immunomodulatory effects have also generated interest in combination with systemic therapies, although this remains investigational.

Nevertheless, evidence remains limited to small institutional series, and standardised treatment protocols are lacking. Current enthusiasm therefore exceeds the available evidence, and these techniques should presently be regarded as investigational rather than standard treatment options.

### 6.4. Radioligand Therapy in Combination with External Beam Radiotherapy (EBRT)

Combination approaches integrating radiotherapy with systemic targeted radionuclide therapies are also gaining interest. In oligometastatic prostate cancer, early studies investigating external beam radiotherapy alongside prostate-specific membrane antigen (PSMA)-targeted radioligand therapy suggest the potential for improved local and distant disease control through simultaneous treatment of visible and microscopic disease [53,54,55,56]. This combined strategy may be particularly attractive for patients with oligopersistent or oligoprogressive disease, where eradication of resistant lesions while maintaining systemic therapy could prolong disease control. However, further research is needed to identify the optimal timing, dosing and indications of this combined therapy [56]. Similar strategies are being explored in neuroendocrine tumours using peptide receptor radionuclide therapy combined with SABR [57].

Although early clinical results are encouraging, the optimal sequencing with external beam radiotherapy, patient selection, toxicity profile, and long-term survival benefit remain uncertain and should be evaluated within prospective clinical trials.

### 6.5. MR-Guided Linear Accelerators (MR Linac)

Technological advances in image guidance are further improving the precision of metastasis-directed radiotherapy. This may become particularly important in oligometastatic disease because patients are surviving long enough to require repeat courses of radiotherapy.

Cone beam CT is the standard image guidance used; however, CT has limited soft tissue contrast, which can make volume delineation challenging [58]. MR-linacs provide superior soft tissue visualisation and enable online adaptive radiotherapy, allowing treatment plans to be modified in real time according to daily anatomical variation [59]. A study by Yang et al. highlighted that in 86.6% of fractions delivered using an MR linac, a clinically significant improvement in target coverage or organ at risk (OAR) avoidance was seen [60]. This improved accuracy supports a reduction in planning target volumes (PTV), which reduces overall dose to organs at risk [61]. This translates into reduced acute and late toxicity alongside improved recovery post-treatment, improving QoL but also enabling patients to be in a position where they can undergo systemic treatment if needed [62]. Furthermore, the reduction in margins offers significant benefit in the case of oligometastatic disease where patients may often have had previous radiotherapy or given improved survival where re-irradiation may be needed in the future [59]. Whilst there is a greater complexity of the treatment workflow through the use of MR linac, Yang et al.’s study of 101 patients showed a median time in the treatment room of 58 min, which was felt to be practical and likely to decrease through streamlining and acclimatisation [60].

Despite its technical advantages, there is currently no high-level evidence demonstrating that MR-guided adaptive radiotherapy improves survival, local control, or patient-reported QoL compared with contemporary CBCT-guided SABR. Furthermore, longer treatment times, increased staffing requirements, workflow complexity, and higher capital costs currently limit widespread implementation. Future studies should establish whether these technical improvements translate into clinically meaningful patient benefit.

Collectively, these technologies illustrate the rapid evolution of metastasis-directed radiotherapy. However, apart from SABR, most remain supported by relatively low- to moderate-level evidence, with few prospective comparative trials demonstrating improvements in survival, toxicity, or QoL. An overview of these techniques and the evidence supporting them is provided in Table 2.

Future research should therefore prioritise randomised studies, cost-effectiveness analyses, incorporation of patient-reported outcomes, and integration of molecular biomarkers to better define which patients derive meaningful benefit. As survival following oligometastatic disease continues to improve, technological innovation must be accompanied by survivorship-focused care models that minimise cumulative toxicity while maintaining long-term QoL.

## 7. Immunotherapy

Increasingly effective systemic therapies have shifted attention towards combination strategies capable of enhancing both local and systemic tumour control. Among these, integration of SABR with immune checkpoint inhibition has generated considerable interest because radiotherapy may augment anti-tumour immunity while immunotherapy may enhance the systemic effects of local radiation. Beyond its direct cytotoxic effects, radiotherapy is now recognised as a potent immunomodulatory treatment capable of enhancing tumour antigen presentation, promoting T-cell priming, stimulating inflammatory cytokine release, and remodelling the tumour microenvironment, thereby potentially augmenting the efficacy of immune checkpoint inhibitors [63,64,65].

One of the most widely discussed mechanisms underpinning this interaction is the abscopal effect, a phenomenon in which localised radiotherapy induces tumour regression at distant, non-irradiated metastatic sites through systemic immune activation. Although historically considered rare, the emergence of checkpoint inhibitors has renewed interest in the clinical relevance of the abscopal effect, with multiple case reports and translational studies suggesting that immunotherapy may enhance both the frequency and magnitude of these responses [66,67,68]. Early prospective studies combining SABR with immune checkpoint blockade have demonstrated encouraging improvements in progression-free survival and systemic disease control across several tumour types, particularly melanoma and non-small cell lung cancer [69]. The phase II PEMBRO-RT trial, for example, demonstrated improved response rates and progression-free survival in patients with metastatic non-small cell lung cancer receiving pembrolizumab following SABR compared with pembrolizumab alone, supporting the biological rationale for this combination, but requiring validation in a randomised phase 3 trial [70,71].

Despite these promising findings, many questions remain regarding the optimal integration of SABR with systemic therapies. The optimal sequencing of radiotherapy and immunotherapy is uncertain, with conflicting evidence regarding the relative benefits of concurrent versus sequential treatment [65,72]. Variability in tumour histology, radiation dose and fractionation, immunotherapy agents, and patient selection likely contribute to the heterogeneity observed across studies, underscoring the need for prospective randomised trials.

As combined modality approaches become increasingly adopted, treatment-related toxicity has emerged as a major consideration, particularly as patients with oligometastatic disease experience prolonged survival [73]. The addition of immunotherapy to SABR has been associated with increased rates of immune-mediated toxicity, including pneumonitis, dermatitis, colitis, hepatitis, endocrinopathies, and neurological complications [74,75,76,77]. The addition of dual immune checkpoint blockade or chemo-immunotherapy may further increase cumulative toxicity through overlapping inflammatory mechanisms and enhanced radiosensitisation [78,79,80].

Interpretation of toxicity data remains challenging because most published evidence originates from retrospective series or early-phase clinical trials with heterogeneous patient populations and relatively short follow-up [81]. Although concerns regarding synergistic toxicity are biologically plausible, current evidence does not consistently demonstrate increased rates of grade ≥3 toxicity compared with immunotherapy alone [76,82,83]. Nevertheless, specific toxicities, including radiation necrosis, pneumonitis, dermatitis, and immune-mediated adverse events, appear to occur more frequently in selected clinical settings, reinforcing the importance of careful patient selection, multidisciplinary management, and vigilant long-term follow-up [83,84,85].

The risk of toxicity also appears to vary according to the immunotherapeutic agent used, with cytotoxic T-lymphocyte-associated protein 4 (CTLA-4) inhibitors generally associated with higher toxicity than programmed death ligand-1 (PD-L1) inhibitors [81,83]. Treatment-related factors, including radiotherapy timing, dose fractionation, and target volume, may also influence toxicity; however, their optimal application remains poorly defined [77,82]. The incorporation of a radiotherapy treatment break may not reduce the risk of side effects due to the sustained immune effects of immunotherapy and may contribute to a loss of tumour control [83]. Recognising these uncertainties, ESTRO and the European Society for Medical Oncology (ESMO) have published consensus recommendations to guide the safe integration of radiotherapy with immunotherapy and other systemic anticancer therapies [81,83]. Future incorporation of predictive biomarkers may further facilitate precision immuno-radiotherapy by identifying patients most likely to benefit from treatment while minimising toxicity.

Despite growing clinical experience, the long-term toxicities associated with combined SABR and immunotherapy remain incompletely understood and represent one of the most important evidence gaps in the management of oligometastatic disease [82]. While acute toxicities are increasingly recognised within prospective trials, survivorship outcomes and patient-reported QoL remain comparatively underreported, particularly in patients surviving many years following treatment [86]. Chronic fatigue, pulmonary fibrosis, neurocognitive impairment, endocrine dysfunction, cardiovascular toxicity, chronic pain, and persistent immune-related adverse events may significantly impact QoL and functional independence [81,87]. Importantly, these toxicities may emerge months or even years following treatment completion, creating challenges for surveillance, attribution, and management.

Future prospective studies should therefore incorporate long-term toxicity monitoring, patient-reported outcomes, and biomarker development to optimise patient selection and guide personalised immuno-radiotherapy strategies.

## 8. Patient Selection and Long-Term Management

Collectively, these evolving technologies continue to shift the boundaries of what is achievable in the radical treatment of advanced disease. However, their successful implementation depends not only on technological advances but also on appropriate patient selection, multidisciplinary decision-making, and structured long-term follow-up. As therapeutic options expand and survival outcomes improve, identifying those patients most likely to benefit from metastasis-directed therapy while avoiding overtreatment has become one of the greatest challenges in modern radiation oncology.

Selection for metastasis-directed therapy requires consideration of multiple interacting clinical, disease-related, biological, and imaging factors. Clinical considerations include performance status, comorbidities, treatment tolerance, life expectancy, and patient preferences and goals of care. Disease-specific factors such as the number, size, and location of metastases, disease-free interval, rate of progression, response to systemic therapy, and control of the primary tumour are also critical determinants of prognosis and treatment suitability. Several retrospective and prospective studies have consistently demonstrated improved outcomes in patients with metachronous disease, prolonged disease-free interval, low metastatic burden, favourable tumour biology, and controlled primary lesions [29,88,89,90,91,92]. Increasingly sensitive imaging modalities and advances in molecular diagnostics are likely to further refine patient selection, although they also introduce uncertainty regarding the optimal management of very low-volume or biologically indolent metastatic disease. Consequently, the expanding use of advanced imaging and ablative therapies has created a growing cohort of patients living with prolonged control of advanced cancer for whom evidence-based follow-up pathways remain poorly defined.

Recognising that a proportion of patients with oligometastatic disease are now treated with long-term disease control or cure as the primary objective, the Palliative Radiotherapy Task Force recently proposed the term Curative Oligometastatic Radiotherapy (CORT) [93]. CORT refers to the delivery of ablative radiotherapy with curative intent to all known sites of disease and aims to distinguish these treatments from conventional palliative radiotherapy. Patients eligible for CORT should meet certain criteria consistent with long-term favourable outcomes, including (but not limited to) a good performance status, favourable tumour histology, limited metastatic disease and, for oligo-recurrent disease, a long disease-free interval before recurrence and metastasis [93].

Clearly outlining the aim of the treatment improves communication with patients and may improve adherence to radiotherapy and acceptance of the risk of toxicity that may be present with ablative doses. This new definition may also improve standardisation of radiotherapy regimes offered for oligometastatic disease between centres. This also enables the collection of comparable data that could be used in clinical trials.

The improved survival seen in patients with metastatic cancer has increasingly shifted cancer towards a chronic disease model [29]. Consequently, there is a growing need to address survivorship, late effects, and long-term QoL, areas that have historically been under-recognised due to the predominant focus on overall survival and progression-free survival outcomes [94]. Patients living with controlled metastatic disease often experience cumulative physical and psychological morbidity arising both from cancer itself and from repeated local and systemic treatments [95]. Chronic fatigue, pain, reduced physical functioning, cognitive difficulties, anxiety surrounding surveillance imaging, financial toxicity, and uncertainty regarding prognosis are increasingly recognised challenges within this patient population.

## 9. Quality of Life

As overall survival improves, QoL has become an equally important endpoint when evaluating metastasis-directed therapies. For many patients with oligometastatic disease, the goal of treatment extends beyond prolonging survival to preserving physical function, independence, cognitive ability, psychological wellbeing, social participation, and the ability to maintain meaningful daily activities. Consequently, treatment success should be evaluated not only by traditional oncological outcomes such as overall survival and progression-free survival, but also by its impact on the patient’s lived experience. Additionally, many of these patients may undergo repeated episodes of local and systemic treatment. Consequently, even relatively modest treatment-related toxicities may accumulate over time and have substantially influence on these patients.

Although long-term QoL data following local ablative interventions remain limited, existing evidence suggests that many patients maintain stable global QoL despite intensive local therapy. For example, in the SABR-5 trial, clinically significant deterioration in quality of life at 36 months was observed in approximately 25% of patients, whereas QoL remained stable or improved in many patients [96]. Similarly, prospective studies evaluating SABR in oligometastatic disease have demonstrated only modest reductions in QoL over time, regardless of treatment approach, with comparable trends also observed in control groups [97,98].

Importantly, preservation of global QoL should not be interpreted as the absence of treatment burden. Global QoL scores may conceal persistent impairments in individual domains including fatigue, pain, physical functioning, cognitive function, emotional wellbeing, sexual health, financial toxicity, and fear of recurrence or progression. These domain-specific outcomes frequently have a greater impact on patients’ everyday lives than overall QoL scores and therefore warrant routine assessment throughout survivorship.

Identifying patients at greatest risk of long-term toxicity and QoL decline remains an important area of ongoing research. Data from the SABR-5 cohort demonstrated that disease progression and adrenal metastases were associated with greater deterioration in patient-reported QoL [99]. Higher-grade toxicities were also associated with poorer quality-of-life outcomes, although statistical significance was not consistently reached, highlighting the need for larger prospective studies with extended follow-up.

Patient-reported outcomes (PROs) have become increasingly important in understanding the true impact of treatment from the patient’s perspective. These outcomes are most commonly captured using validated patient-reported outcome measures (PROMs), which provide a standardised and reliable method of assessing symptoms, functional status, health-related quality of life, and other aspects of patient well-being over time. While clinician-reported toxicity grading remains essential, healthcare professionals frequently underestimate the severity, frequency, and impact of symptoms experienced by patients. PROs provide (complementary) information that cannot be fully captured through clinician assessment. Incorporating the patient’s perspective into routine care through the use of validated PROMs facilitates more patient-centred treatment decisions and allows clinicians to better balance oncological benefit against treatment-related burden. In the setting of oligometastatic disease, where patients may experience prolonged survival following repeated local and systemic therapies, routine PROM-based PRO assessment offers an opportunity to monitor the cumulative effects of treatment and guide personalised survivorship care.

The integration of PROMs and the PRO data they generate into routine clinical practice also aligns closely with the principles of supportive oncology, which emphasise proactive identification and management of physical, psychological, functional, and social concerns throughout the disease trajectory rather than responding only after symptoms become clinically apparent. Longitudinal PRO monitoring may therefore help identify patients requiring support before deterioration becomes irreversible, facilitate timely referral to rehabilitation and supportive care services, and evaluate the effectiveness of these interventions over time. Additionally, a better understanding of predictors for long-term toxicity and reduced QoL would improve patient selection, informed consent, survivorship planning, and post-treatment surveillance strategies.

Future clinical trials evaluating metastasis-directed therapies should therefore incorporate PROs as key endpoints alongside survival, local control, and toxicity. As technological advances continue to improve oncological outcomes, demonstrating that these gains translate into meaningful improvements in patients’ daily lives will become equally important. Routine integration of PROMs into both prospective studies and clinical practice will be essential to optimise survivorship care and ensure that treatment success is measured not only by how long patients live, but also by how well they live.

## 10. Supportive Oncology

The increasing use of metastasis-directed therapies, together with improvements in systemic treatment, has created a growing population of patients living for prolonged periods with controlled metastatic disease. Consequently, supportive oncology has become an essential component of modern oligometastatic care, with increasing emphasis on preserving long-term health, functional independence, and QoL alongside optimising oncological outcomes.

Supportive oncology is an emerging multidisciplinary specialty focused on the prevention and management of the physical, psychological, social, and functional consequences of cancer and its treatment across the entire disease trajectory [100]. Unlike traditional models that closely associate supportive care with end-of-life management, contemporary supportive oncology extends from diagnosis through survivorship and includes patients receiving curative, life-prolonging, and palliative treatments. Increasing evidence demonstrates that supportive oncology interventions not only improve symptom control but also preserve physical function, treatment tolerance, and patient-reported QoL, supporting their integration alongside active cancer treatment rather than reserving them for patients with advanced symptoms [101]. Its focus has therefore shifted from reactive symptom management to proactive preservation of health, prevention of treatment-related morbidity, rehabilitation, and survivorship. This evolution is increasingly reflected in national cancer strategies. For example, the 2026 National Cancer Plan for England identifies expansion of supportive oncology and personalised survivorship services as key priorities for improving outcomes for people living with and beyond cancer [102].

The concept of “living with and beyond cancer” has become increasingly relevant within oncology and reflects the growing number of patients surviving for prolonged periods following cancer diagnosis, including those with metastatic disease [103]. Globally, this population is estimated to be growing at a rate of 2% each year [104,105]. These patients frequently experience ongoing uncertainty regarding prognosis, repeated cycles of surveillance imaging and treatment, cumulative toxicity burden, financial stress, altered social roles, and persistent fear of progression or recurrence [94]. This concept is particularly relevant to oligometastatic disease, where repeated episodes of surveillance, metastasis-directed therapy, and systemic treatment increasingly resemble the management of a chronic condition rather than a traditional trajectory towards cure or end-of-life care.

**Figure 2:**
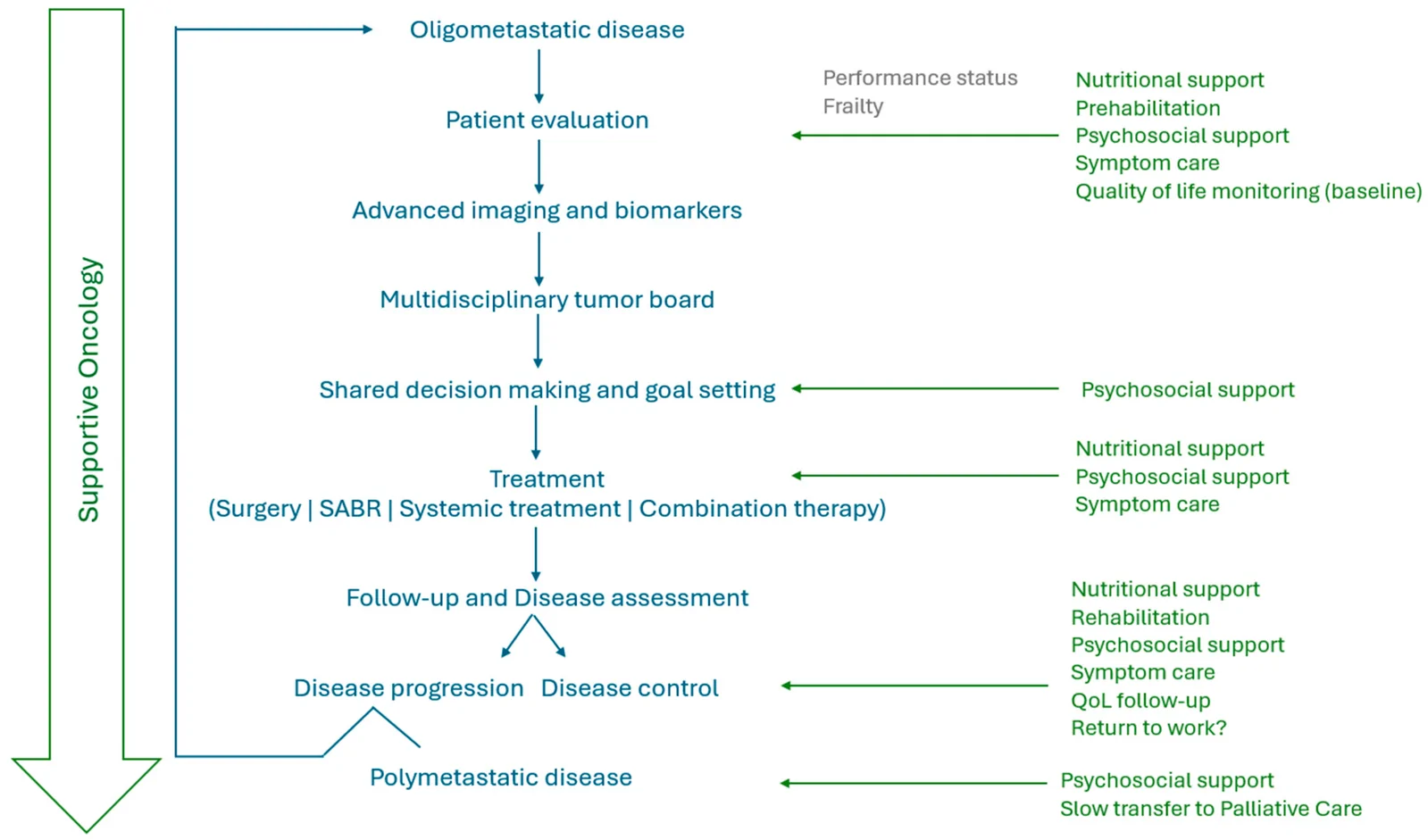
Proposed Integrated Supportive Oncology Pathway for Patients with Oligometastatic Disease.

Consequently, survivorship can no longer be viewed solely as a post-curative concept confined to early-stage disease but must increasingly incorporate patients living with durable control of metastatic cancer. Cancer survivorship has itself been broadly defined by the National Coalition for Cancer Survivorship (NCCS) as beginning at diagnosis and continuing through the remainder of life, encompassing the physical, psychosocial, and economic effects of cancer experienced by patients, families, and caregivers [106]. However, despite this broad definition, survivorship services have historically focused on patients treated with curative intent, leading to a relative absence of structured survivorship pathways for patients with advanced or oligometastatic disease. In response to this deficiency, the Multinational Association of Supportive Care in Cancer (MASCC) and the American Society of Clinical Oncology (ASCO) published recommendations in 2024 identifying key standards for survivorship for those living with advanced or metastatic cancer [107]. These recommendations advocate risk-stratified survivorship care incorporating routine PROMs, multidisciplinary rehabilitation, psychosocial assessment, exercise and nutritional interventions, coordinated follow-up, and improved communication between oncology specialists, primary care, and supportive oncology services. Such frameworks provide an important foundation for developing survivorship pathways specifically tailored to patients with oligometastatic disease. Figure 2 demonstrates a proposed integrated Supportive Oncology Pathway for patients with oligometastatic disease, emphasising the continued presence during the disease trajectory.

Approximately 8% of the cancer population in England are living with treatable but incurable cancer, and this number is only set to rise with continued advances in treatment regimens [108,109]. The integration of supportive oncology within radiotherapy services is therefore becoming increasingly important. Radiation oncologists are uniquely positioned to coordinate many aspects of survivorship because they frequently maintain longitudinal relationships with patients undergoing repeated courses of SABR or other local therapies. Supportive oncology should therefore be embedded throughout radiotherapy pathways, incorporating prehabilitation, routine collection of PROMs, exercise medicine, nutritional optimisation, fatigue management, psychological support, sexual health services, cognitive rehabilitation, vocational support, and personalised survivorship care planning. Follow-up should ideally involve coordinated multidisciplinary input from radiation oncologists, medical oncologists, supportive oncology specialists, rehabilitation professionals, psychologists, primary care physicians, and allied health professionals, reflecting the complex needs of long-term survivors of metastatic cancer. Emerging evidence suggests that early integration of supportive oncology alongside active oncological treatment improves symptom burden, patient satisfaction, QoL, and potentially even survival outcomes in advanced cancer populations [110,111,112,113,114]. It should be noted, however, that although randomised evidence supporting exercise, prehabilitation, and multidisciplinary rehabilitation has expanded considerably across oncology, high-quality data specific to patients with oligometastatic disease remain limited, as patients treated with metastasis-directed therapies such as SABR have largely been excluded from these studies. As advances in SABR and systemic therapy continue to extend survival, prospective randomised trials evaluating exercise, rehabilitation, and survivorship interventions specifically in the oligometastatic population are urgently needed. Establishing evidence-based rehabilitation strategies represents an important research priority in supportive oncology for patients living with controlled metastatic disease.

Despite rapid advances in metastasis-directed therapies, models of survivorship care for patients living with durable control of metastatic cancer remain poorly defined [115,116]. In many healthcare systems, there is limited clarity regarding who is responsible for coordinating long-term survivorship care. Follow-up may variably fall to the treating radiation oncologist, medical oncologist, supportive oncology team, primary care physician, or combinations thereof, often resulting in fragmented care and unmet supportive needs. The increasing complexity of modern cancer survivorship raises important questions regarding whether dedicated survivorship or supportive oncology services for patients with advanced cancer should evolve into a more formally recognised subspecialty within oncology care. Development of dedicated survivorship clinics for patients with controlled metastatic disease may provide one solution by integrating surveillance, toxicity monitoring, rehabilitation, psychosocial care, and coordination between oncology and primary care services. Such clinics could also facilitate systematic assessment of late toxicities, longitudinal PROM collection, personalised rehabilitation, and prospective evaluation of survivorship interventions, thereby generating the evidence needed to refine future models of care. Supportive oncology should therefore become an integral component of oligometastatic care rather than an adjunct introduced only after treatment-related problems arise.

To date, no evidence-based survivorship pathway has been developed specifically for patients with oligometastatic disease. Nevertheless, several core components consistently emerge from survivorship recommendations for advanced cancer, supportive oncology, rehabilitation, and PRO monitoring and coordinated care between oncology specialists, primary care physicians, and allied health professionals. Future prospective studies should evaluate whether such integrated models improve quality of life, functional independence, healthcare utilisation, and long-term oncological outcomes in this growing patient population.

Importantly, supportive oncology should not be regarded as synonymous with palliative care. Although the disciplines overlap in symptom control and psychosocial support, supportive oncology provides a broader framework encompassing rehabilitation, optimisation of function, survivorship planning, exercise medicine, nutritional support, vocational rehabilitation, sexual health, and management of chronic treatment-related toxicities across the entire disease continuum. Additionally, many patients with durable control of metastatic disease do not perceive themselves to be approaching the end of life and may regard referral to palliative care as inconsistent with their lived experience. Their priorities frequently centre on maintaining employment, preserving physical function, sustaining relationships, managing chronic treatment-related toxicities, and coping with uncertainty regarding future disease progression. These evolving needs reinforce the case for dedicated supportive oncology pathways that complement rather than replace specialist palliative care. As survival continues to improve, development of disease-specific survivorship models for oligometastatic disease should become a research and service priority, ensuring that advances in cancer treatment are matched by equivalent advances in long-term supportive care.

## 11. Future Directives

Future directions in this field should focus on the development of evidence-based survivorship pathways specifically designed for patients with oligometastatic and chronic metastatic cancer. Prospective survivorship studies incorporating PROs, late toxicity monitoring, functional assessments, and psychosocial measures are urgently needed to better characterise the long-term consequences of increasingly intensive multimodality treatment approaches. Greater integration of supportive oncology within radiotherapy departments and multidisciplinary metastatic services may help ensure that improvements in survival are matched by improvements in long-term QoL, functional independence, and patient-centred outcomes. Development of consensus survivorship pathways and prospective evaluation of multidisciplinary care models should be considered a research priority. Future studies should incorporate patient-reported outcomes, functional measures, return-to-work outcomes, health economic analyses, and long-term toxicity assessment alongside traditional oncological endpoints.

Ultimately, prospective evaluation of integrated survivorship models, such as the conceptual supportive oncology pathway proposed in this review, will be essential to determine whether coordinated multidisciplinary care improves patient outcomes, healthcare utilisation, and long-term quality of life.

Alongside improvements in survivorship care, advances in biomarker research are likely to play an increasingly important role in refining patient selection, treatment adaptation, and response evaluation in oligometastatic disease.

Research into circulating tumour DNA (ctDNA), circulating tumour cells, tumour mutational burden, immune profiling, radiomics, and molecular signatures may help identify patients most likely to benefit from metastasis-directed therapies while distinguishing biologically indolent oligometastatic disease from occult polymetastatic disease. Biomarkers may also facilitate earlier detection of minimal residual disease, prediction of treatment response, and identification of patients at increased risk of recurrence or long-term toxicity. Integration of liquid biopsy approaches into post-treatment surveillance strategies may enable more personalised follow-up pathways and earlier intervention prior to radiological progression [117,118].

Artificial intelligence (AI) and machine learning are also expected to transform multiple aspects of oligometastatic cancer management.

AI-driven imaging analysis and radiomics models are increasingly being evaluated in prospective multicentre studies for automated target delineation, lesion detection, treatment adaptation, toxicity prediction, and prognostication [119]. Although early results are encouraging, external validation, regulatory approval, integration into clinical workflows, and demonstration of clinical benefit remain necessary before widespread implementation.

Within radiotherapy planning, AI has the potential to improve workflow efficiency, standardise contouring, optimise adaptive radiotherapy strategies, and facilitate increasingly personalised treatment planning [120]. Machine learning algorithms integrating imaging, genomic, clinical, and patient-reported data have considerable potential to improve patient selection; however, most models remain retrospective and require prospective validation across diverse patient populations.

AI may additionally contribute to survivorship and follow-up care through predictive modelling of recurrence risk, automated toxicity monitoring, and integration of remote symptom reporting platforms [121]. Importantly, these technologies should not only improve treatment delivery but also facilitate more personalised survivorship care by enabling earlier detection of treatment-related complications, more efficient longitudinal monitoring, and proactive supportive care interventions> As patients with advanced cancer increasingly transition into long-term survivorship, digital health technologies and AI-supported surveillance systems may help deliver more individualised and resource-efficient follow-up strategies while improving early detection of treatment-related complications and disease progression [122].

Beyond advances in radiotherapy and AI, other precision oncology strategies, including nanoparticle-based drug delivery systems and theranostic approaches, are also being actively explored across oncology. Although these technologies remain largely investigational and are currently more relevant to selected disease sites such as primary and secondary brain tumours, they illustrate the broader trend towards increasingly personalised, multimodal cancer treatment. As with emerging radiotherapy technologies, careful prospective clinical validation is required to demonstrate meaningful improvements in survival, toxicity, and PROs before widespread implementation [123].

Collectively, these developments illustrate that the future of oligometastatic care will extend far beyond technological innovation alone. Advances in imaging, biomarkers, artificial intelligence, and novel therapeutics must be accompanied by equivalent progress in survivorship care, supportive oncology, and multidisciplinary service delivery to ensure that patients benefit not only from longer survival, but also from better long-term health and quality of life.

## 12. Overall Perspective

The rapid evolution of oligometastatic cancer from a uniformly palliative condition to one in which long-term disease control is increasingly achievable represents one of the most important paradigm shifts in modern oncology. This transformation has fundamentally altered the needs of this patient population, creating a growing cohort of long-term survivors who face unique physical, psychological, functional, and social challenges that extend well beyond completion of treatment.

While remarkable progress has been made in imaging, radiotherapy, systemic therapies, and patient selection, advances in supportive care have not kept pace with these technological developments. As a result, patients frequently transition from intensive cancer treatment into survivorship without structured pathways to address chronic toxicities, rehabilitation needs, psychosocial wellbeing, or long-term quality of life.

This review therefore argues that the next major advance in oligometastatic cancer care should not be technological innovation alone, but the integration of evidence-based survivorship care into routine multidisciplinary practice. The conceptual Integrated Supportive Oncology Pathway proposed in this review is intended as a framework to stimulate discussion, future research, and the development of dedicated survivorship models for patients with oligometastatic disease.

## 13. Conclusions

The management of oligometastatic disease has evolved from a predominantly palliative paradigm to one in which carefully selected patients may achieve durable disease control and, in some cases, long-term remission or cure through metastasis-directed therapies. This paradigm shift has fundamentally changed the goals of treatment, extending beyond tumour control alone to encompass long-term survivorship, preservation of function, and QoL.

Advances in SABR, molecular imaging, biomarker research, immunotherapy, MR-guided adaptive radiotherapy, proton therapy, artificial intelligence, and emerging radiotherapy technologies continue to expand treatment possibilities while simultaneously increasing the complexity of patient selection and long-term survivorship care.

Although SABR is now supported by randomised evidence in selected patient populations, many emerging technologies, including proton therapy, MR-guided adaptive radiotherapy, FLASH radiotherapy, radioligand combinations, and AI-assisted approaches, remain supported primarily by early-phase, retrospective, or dosimetric evidence. Their integration into routine practice should therefore be guided by prospective evaluation demonstrating meaningful improvements in survival, toxicity, QoL, and cost-effectiveness.

A central message of this review is that technological innovation, while essential, is insufficient on its own. Equally important is accurate patient selection by integrating tumour biology, advanced imaging, clinical characteristics, and molecular biomarkers to identify those patients most likely to benefit from metastasis-directed therapies while avoiding overtreatment.

Improvements in survival must be accompanied by structured survivorship pathways that integrate multidisciplinary supportive oncology, rehabilitation, systematic and standardised PRO monitoring, psychosocial support, and coordinated long-term follow-up. Development of evidence-based survivorship models specifically for patients with oligometastatic disease represents one of the major unmet needs in contemporary oncology and should become a priority for future research. Future prospective studies should extend beyond traditional oncological endpoints by incorporating long-term PROs, functional recovery, rehabilitation, late toxicity, return-to-work, health economic evaluation, and multidisciplinary models of survivorship care.

Ultimately, the success of modern radiation oncology should no longer be judged solely by improvements in local control or survival. As increasing numbers of patients with oligometastatic disease live for many years following metastasis-directed therapy, equal emphasis must be placed on preserving function, QoL, psychosocial wellbeing, and long-term independence. We believe that the oligometastatic paradigm is now transitioning from a treatment-focused model towards a survivorship-focused model. Clinical research, healthcare systems, and multidisciplinary cancer services must evolve accordingly to ensure that advances in cancer treatment are matched by equivalent advances in long-term supportive care. Only by combining technological innovation with evidence-based survivorship care will we fully realise the promise of modern radiation oncology: not merely helping patients live longer, but enabling them to live better.

## Figures and Tables

**Table 1:** Overview of the stereotactic ablative radiotherapy studies mentioned in this review. NSCLC: non-small cell lung cancer, SABR: stereotactic ablative radiotherapy, QoL: quality of life. Please note that is not a complete overview of trials that include SABR.

Trial	Tumour Type	Patient Population	Intervention	Key Findings	Clinical Significance
***SABR-COMET*** (Palma et al., 2019; *2020 follow-up*) [30,31]	Mixed tumour types	Controlled primary tumour; 1–5 metastases	Standard of care ± SABR to all lesions	Improved overall survival (5-year OS 42% vs. 17%), progression-free survival, and durable local control, with acceptable toxicity	First randomised phase II trial demonstrating survival benefit of SABR across multiple tumour types; established proof-of-principle for metastasis-directed therapy
***ORIOLE*** (Phillips et al., 2020) [32]	Prostate cancer	Recurrent hormone-sensitive oligometastatic disease (≤3 metastases)	Observation vs. SABR	Significantly reduced disease progression at 6 months; improved progression-free survival; lower development of new metastases	Demonstrated the benefit of SABR in delaying progression and highlighted the value of advanced imaging for patient selection
***STOMP*** (Ost et al., 2018, 2020; *5-year update*) [34,35]	Prostate cancer	Oligorecurrent hormone-sensitive disease (≤3 metastases)	Surveillance vs. metastasis-directed therapy (SABR or surgery)	Prolonged androgen deprivation therapy (ADT)-free survival and improved progression-free survival	Supported metastasis-directed therapy to delay systemic treatment and preserve QoL
**Gomez et al.** (*2016; 2019 update*) [33,36]	NSCLC	Stage IV NSCLC without progression after first-line systemic therapy; ≤3 metastases	Local consolidative therapy (radiotherapy or surgery) vs. maintenance/observation	Significant improvements in progression-free survival and overall survival	Established consolidative local therapy as standard consideration in selected oligometastatic NSCLC
***SABR-5*** (Olson et al.) [37]	Mixed tumour types	1–5 metastases	SABR	Maintained global QoL; approximately 25% experienced clinically significant QoL deterioration at 36 months	Demonstrated that aggressive local treatment can achieve durable disease control while maintaining long-term QoL

**Table 2:** Overview of novel treatments.

Technology	Principle/Clinical Role	Potential Advantages	Current Limitations	Current Level of Evidence
Proton beam therapy	Delivers radiation using charged particles with a Bragg peak, reducing exit dose to surrounding tissues.	Improved normal tissue sparing; may reduce cumulative toxicity; advantageous for lesions adjacent to critical organs or in re-irradiation.	High capital cost, limited availability, reimbursement challenges, lack of prospective trials demonstrating superiority over photon SABR.	**Moderate.** Retrospective studies and dosimetric analyses; prospective comparative evidence remains limited.
FLASH radiotherapy	Ultra-high dose rate irradiation (>40 Gy/s) designed to spare normal tissues while maintaining tumour control.	Potential reduction in acute and late toxicity; improved therapeutic ratio; may facilitate dose escalation.	Clinical experience remains extremely limited; uncertainties regarding treatment planning, quality assurance, long-term safety, and reproducibility.	**Low.** Predominantly preclinical data with limited early-phase clinical studies.
GRID/Lattice radiotherapy	Spatially fractionated radiotherapy delivering heterogeneous high-dose regions within bulky tumours.	May improve treatment of bulky lesions, re-irradiation, or tumours near critical organs; possible immunomodulatory effects.	Small institutional series only; lack of standardised protocols and prospective comparative trials.	**Low.** Early clinical experience; investigational.
Radioligand therapy + EBRT	Combines targeted radionuclide therapy with external beam radiotherapy to treat macroscopic and microscopic disease.	Potentially improves local and systemic disease control; attractive for oligoprogressive and oligopersistent disease.	Optimal sequencing, patient selection, toxicity, and long-term outcomes remain uncertain.	**Low–Moderate.** Early clinical studies, mainly in prostate cancer.
MR-guided adaptive radiotherapy (MR-linac)	Real-time MRI guidance with online adaptive replanning.	Superior soft tissue visualisation; reduced treatment margins; improved target coverage; potential reduction in toxicity; facilitates re-irradiation and adaptive dose delivery.	Resource intensive; prolonged treatment times; limited availability; specialist training required; comparative outcome data remain limited.	**Moderate.** Growing prospective institutional experience; randomised evidence lacking.

EBRT: external beam radiotherapy.

## Data Availability

No new data were created or analyzed in this study. Data sharing is not applicable to this article.

## Abbreviations

The following abbreviations are used in this manuscript:

AI	Artificial Intelligence
ASCO	American Society of Clinical Oncology
CORT	Curative oligometastatic radiotherapy
CTDNA	Circulating tumour DNA
CTLA-4	Cytotoxic T-lymphocyte-associated protein 4
EBRT	External beam radiotherapy
EORTC	European Organisation for Research and Treatment of Cancer
ESMO	European Society for Medical Oncology
ESTRO	European Society for Radiotherapy and Oncology
MASCC	Multinational Association of Supportive Care in Cancer
MR linac	MR-guided linear accelerators
OAR	Organ at risk
NCCS	National Coalition for Cancer Survivorship
PDL-1	Programmed cell death ligand 1
PRO	Patient Reported Outcome
PROM	Patient Reported Outcome Measure
PSMA	Prostate-specific membrane antigen
PTV	Planning target volumes
QoL	Quality of Life
SABR	Stereotactic ablative radiotherapy
